# Editorial: Improving Animal Welfare through Genetic Selection

**DOI:** 10.3389/fgene.2016.00069

**Published:** 2016-04-26

**Authors:** Wendy M. Rauw

**Affiliations:** Departamento de Mejora Genética Animal, Instituto Nacional de Investigación y Tecnología Agraria y AlimentariaMadrid, Spain

**Keywords:** selection, genetic, welfare, livestock production, livestock improvement, sustainable agriculture, animal breeding, genetics

The Food and Agriculture Organization of the United Nations predicts that the projected massive global increase in demand for livestock products will continue for several decades. According to Delgado et al. (1999), it is appropriate to term the course of these events a “Livestock Revolution,” which, as opposed to the Green Revolution, is driven by demand. While more precise production technologies, nutrition, and genetic selection methodologies will be successful in reducing the “yield gap,” the production is limited by finite resources including land, water, and energy, thus emphasizing the need for intensification. However, this often requires additional fertilizer, water, and chemical use (Foley, 2011). Godfray et al. (2010), thus, wrote: “A threefold challenge now faces the world: Match the rapidly changing demand for food from a larger and more affluent population to its supply; do so in ways that are environmentally and socially sustainable; and ensure that the world's poorest people are no longer hungry.”

Intensification of livestock production in particular includes an important additional factor to the sustainability equation: the living animal. In response to the morality of intensive livestock production, the last few decades have witnessed a greater consumer demand for organic foods and free range products, and an increased political response and research toward animal welfare issues, particularly driven by public opinion. In addition, continuous selection for high production in livestock has resulted in animals that have been shown to be more at risk for behavioral, physiological, and immunological problems. For example, in this issue, Canario et al. showed that modern 1998-type French Large White sows with high lean growth rate and prolificness at birth were less active in the first 6 h after birth and less attentive to piglets, resulting in a higher risk of piglet death than 1977-type sows. As Van Rooijen indicated, suffering may result from a loss of harmony in animals with themselves (their physiology) and with their environment (natural environment vs. intensive production systems). Therefore, it is unlikely that further intensification of livestock production practices can count on much public acceptance if no measures are taken to guarantee sustainability. “Sustainable intensification” of livestock must be defined by economic profitability through improvement of productive output, while maintaining animal health and welfare, and without compromising environmental resources during the production process. Livestock breeding programs of today and of the future must adhere to this definition; therefore, animals must be bred that are robust.

Robustness may be improved through the use of reaction norms analysis (as reviewed by Rauw and Gomez Raya) and through the inclusion of robustness traits in the breeding objective. The last few decades have seen the inclusion of functional traits such as those related to longevity, health, and fertility, in addition to production traits in selection indexes. Indeed, these traits have a clear economic value and are considered as indicators of well-being. In this issue, Strucken et al. reviewed the genetics that underlies the complex physiological dynamics behind the lactation cycle of dairy cattle as a new potential functional trait. Selection for a production curve that allows production without inducing an energy deficiency, by distributing the total quantity of milk per lactation more equally over time, could improve health and welfare. Kassahun et al. described admixture mapping as an approach for gene discovery of economically and medically important traits. Their work describes the potential of admixture mapping in hybrid domestic animals with divergent ancestral genomes derived from *Bos taurus* and *Bos indicus*, to search for genomic regions associated with susceptibility to bovine tuberculosis—a chronic respiratory infection in cattle.

In addition to the inclusion of functional traits, several authors discuss the feasibility of including behavioral traits in the selection criteria. For example, in this issue, Haskell et al. extensively reviewed the feasibility of including temperament traits in dairy and beef cattle selection indices. This has a clear economic value through the associations between temperament and productivity; in addition, animals that respond poorly to handling suffer negative emotional and physical experiences, resulting in reduced welfare. Including behavioral traits in the selection criteria pose a number of challenges. For example, as extensively described by Ellen et al., when animals are kept in groups, social interactions can have large positive (cooperation and mothering behavior) and negative (competition and aggression) effects on individual welfare, productivity, and health. As a result, response to selection using classical selection methods for socially affected traits may not always be optimal. Alternatively, statistical methods have been derived that capture the total genetic variation underlying a trait by taking into account both the direct genetic effect of an individual and its social genetic effect on the phenotype of its group mates. The theoretical and empirical works on social genetic effects in livestock and the application and implication of its inclusion in livestock breeding programs are extensively reviewed by Ellen et al. Selection programs to improve associative effects or social impacts of one animal on the performance of another in poultry are described by Muir et al. The authors indicate that breeding programs that involve multi-level selection, and multi-trait selection methods where one of the traits includes indirect genetic effects, will improve both production traits and animal well-being at the same time.

In 2012, the Farm Animal Welfare Council concluded that farm animal breeding companies should be congratulated for the progress made on breeding goals aimed at improving robustness and health and welfare traits. However, there are still some issues associated with high production levels resulting in poor animal welfare. With this research topic, and thanks to the generous willingness of all participants to contribute, we aimed to present examples that show that research is devoted to improve welfare in livestock through selection, which will enhance sustainability of livestock production systems in the future.

## Author contributions

The author confirms being the sole contributor of this work and approved it for publication.

### Conflict of interest statement

The author declares that the research was conducted in the absence of any commercial or financial relationships that could be construed as a potential conflict of interest.
